# Supplementary material for: The Systems Biology Research Tool: evolvable open-source software
Source: BMC Syst Biol. 2008 Jun 29;2:55. doi: 10.1186/1752-0509-2-55 (PMC2446383; doi:10.1186/1752-0509-2-55)
Supplement: Additional file 1 — SBRT Archive. An archive of the current version of the Systems Biology Research Tool. [file 1752-0509-2-55-S1.zip › sbrt-1.4.0/doc/developers_guide/api/index-files/index-16.html]

P-Index


|  |  |  |
| --- | --- | --- |
| **Overview** \| Package \| Class \| Use \| **Tree** \| **Deprecated** \| **Index** \| **Help** | |  |
| **PREV LETTER**   **NEXT LETTER** | **FRAMES**    **NO FRAMES**     **All Classes** |  |


A B C D E F G H I J K L M N O P Q R S T U V W X Y Z 

---


## **P**

**P\_MAX** - Static variable in class sbrt.shell.mng.fba.InitialPointManager: The keyword used to denote the P intercept (the value of positioning variables at the edge of the convex poytope). **P\_VECTOR\_FILE\_NAME** - Static variable in class sbrt.shell.mng.fba.InitialPointManager: The keyword used to denote the name of the C vector output file. **PalssonRxnNodeParser** - Class in sbrt.shell.text.fba: This class is used to make a direct translation from SBML 'reaction' nodes into Systems Biology Research Tool objects. **PalssonRxnNodeParser()** - Constructor for class sbrt.shell.text.fba.PalssonRxnNodeParser: Constructs a new SBML reaction node parser. **PalssonSbmlReader** - Class in sbrt.shell.proc.fba: This class is used to read SBML files downloaded directly from Palsson's website. **PalssonSbmlReader(String)** - Constructor for class sbrt.shell.proc.fba.PalssonSbmlReader: Constructs a new SBML file reader. **PalssonSbmlReaderManager** - Class in sbrt.shell.mng.fba: This class is used to manage the reading of SBML files obtained from Palsson's website. **PalssonSbmlReaderManager()** - Constructor for class sbrt.shell.mng.fba.PalssonSbmlReaderManager: **PalssonSbmlTranslater** - Class in sbrt.shell.proc.fba: This class is used to translate SBML files from Palsson's website into the Systems Biology Research Tool's *FBA Reaction Files* and *FBA Catalyst Files*. **PalssonSbmlTranslater()** - Constructor for class sbrt.shell.proc.fba.PalssonSbmlTranslater: Constructs a new SBML translater. **PalssonSbmlTranslaterManager** - Class in sbrt.shell.mng.fba: This class is used to manage the translation of SBML files from Palsson's website into the Systems Biology Research Tool's *FBA Reaction Files* and *FBA Catalyst Files*. **PalssonSbmlTranslaterManager()** - Constructor for class sbrt.shell.mng.fba.PalssonSbmlTranslaterManager: **ParallelHyperplanes**<V> - Class in sbrt.kernel.math.geom: This class is used to represent a pair of bounding parallel hyperplanes. **ParallelHyperplanes(LinearComb<V>, Interval)** - Constructor for class sbrt.kernel.math.geom.ParallelHyperplanes: Constructs a new pair of bounding parallel hyperplanes. **PARAM\_FILE** - Static variable in class sbrt.shell.ProgramSolverFactory: The keyword used to denote a parameter file for a program solver. **parse(String)** - Method in class sbrt.shell.text.AbstractLinkedHashSetFormat: Parses the provided string and returns its corresponding collection of elements as a `LinkedHashSet`. **parse(String)** - Method in class sbrt.shell.text.AbstractListFormat: Parses the provided string and returns its corresponding collection of elements as a list. **parse(String)** - Method in interface sbrt.shell.text.BooleanFormat: Parses the provided string and returns its corresponding boolean value. **parse(String)** - Method in class sbrt.shell.text.BoolRelFormatV1: Parses the provided string and returns its corresponding collection of elements as a `LinkedHashSet`. **parse(String)** - Method in class sbrt.shell.text.chem.CatalystFormatV1: Parses the provided string and returns its corresponding catalyst name. **parse(String)** - Method in class sbrt.shell.text.chem.CatalystFormatV2: Parses the provided string and returns its corresponding catalyst name. **parse(String)** - Method in class sbrt.shell.text.chem.ChemSpeciesFormatV1: Parses the provided string and returns its corresponding species name. **parse(String)** - Method in class sbrt.shell.text.chem.IrrevRxnFormatV1: Parses the provided string and returns its corresponding irreversible reaction. **parse(String)** - Method in class sbrt.shell.text.chem.IrrevRxnFormatV2: Parses the provided string and returns its corresponding irreversible reaction. **parse(String)** - Method in class sbrt.shell.text.chem.RevRxnFormatV1: Parses the provided string and returns its corresponding chemical reaction. **parse(String)** - Method in class sbrt.shell.text.chem.RevRxnFormatV2: Parses the provided string and returns its corresponding chemical reaction. **parse(String)** - Method in class sbrt.shell.text.chem.RxnNameFormatV1: Parses the provided string and returns its corresponding reaction name. **parse(String)** - Method in interface sbrt.shell.text.CollectionFormat: Parses the provided string and returns its corresponding collection of elements. **parse(String)** - Method in interface sbrt.shell.text.DoubleFormat: Parses the provided string and returns its corresponding double precision number. **parse(String)** - Method in class sbrt.shell.text.DoubleFormatV1: Parses the provided string and returns its corresponding double precision number. **parse(String)** - Method in class sbrt.shell.text.DoubleFormatV2: Parses the provided string and returns its corresponding double precision number. **parse(String)** - Method in class sbrt.shell.text.fba.BiggBracketParserV1: Parses the provided BiGG style bracketed expression. **parse(String)** - Method in interface sbrt.shell.text.fba.CatalystListFormat: Parses the provided string and returns the list of catalyst names it contains. **parse(String)** - Method in class sbrt.shell.text.fba.CatalystListFormatV1: Parses the provided string and returns the list of catalyst names it contains. **parse(String)** - Method in interface sbrt.shell.text.fba.CatalystSetFormat: Parses the provided string and returns the set of enzyme names it contains. **parse(String)** - Method in class sbrt.shell.text.fba.CatalystSetFormatV1: Parses the provided string and returns the set of catalyst names it contains. **parse(String)** - Method in class sbrt.shell.text.fba.CatalystVerifier: Parses the provided string and returns its corresponding catalyst name. **parse(String)** - Method in class sbrt.shell.text.fba.ChemSpeciesVerifier: Parses the provided string and returns its corresponding chemical species. **parse(String)** - Method in class sbrt.shell.text.fba.FbaOptHeaderParserV1: Parses the provided string and returns its corresponding set of FBA optimization headers as a `LinkedHashSet`. **parse(String)** - Method in class sbrt.shell.text.fba.IrrevRxnFormatV1: Parses the provided string and returns its corresponding chemical reaction. **parse(String)** - Method in class sbrt.shell.text.fba.RxnNameExprFormatV1: Parses the provided string and returns its corresponding linear combination of reaction names. **parse(String)** - Method in interface sbrt.shell.text.fba.RxnNameListFormat: Parses the provided string and returns the list of reaction names it contains. **parse(String)** - Method in class sbrt.shell.text.fba.RxnNameListFormatV1: Parses the provided string and returns the list of reaction names it contains. **parse(String)** - Method in class sbrt.shell.text.fba.RxnNameOrExprFormatV1: Parses the provided string and returns its corresponding object. **parse(String)** - Method in interface sbrt.shell.text.fba.RxnNameSetFormat: Parses the provided string and returns the set of reaction names it contains. **parse(String)** - Method in class sbrt.shell.text.fba.RxnNameSetFormatV1: Parses the provided string and returns its corresponding collection of reaction names as a `LinkedHashSet`. **parse(String)** - Method in class sbrt.shell.text.fba.RxnNameVerifier: Parses the provided string and returns its corresponding reaction name. **parse(String)** - Method in class sbrt.shell.text.FileFormatFormat: Parses the provided string and returns its corresponding file format. **parse(String)** - Method in interface sbrt.shell.text.IntegerFormat: Parses the provided string and returns its corresponding integer. **parse(String)** - Method in class sbrt.shell.text.IntegerFormatV1: Parses the provided string and returns its corresponding integer. **parse(String)** - Method in interface sbrt.shell.text.LinkedHashSetFormat: Parses the provided string and returns its corresponding collection of elements as a `LinkedHashSet`. **parse(String)** - Method in interface sbrt.shell.text.ListFormat: Parses the provided string and returns its corresponding collection of elements as a list. **parse(String)** - Method in interface sbrt.shell.text.LongFormat: Parses the provided string and returns its corresponding long. **parse(String)** - Method in class sbrt.shell.text.LongFormatV1: Parses the provided string and returns its corresponding long. **parse(String)** - Method in class sbrt.shell.text.math.BoundedIntervalFormatV1: Parses the provided string and returns its corresponding interval. **parse(String)** - Method in interface sbrt.shell.text.math.CoeffFormat: Parses the provided string and returns its corresponding coefficient. **parse(String)** - Method in class sbrt.shell.text.math.CoeffFormatV1: Parses the provided string and returns its corresponding coefficient. **parse(String)** - Method in class sbrt.shell.text.math.CoeffFormatV2: Parses the provided string and returns its corresponding coefficient. **parse(String)** - Method in class sbrt.shell.text.math.DirEdgeFormatV1: Parses the provided string and returns its corresponding directed edge. **parse(String)** - Method in class sbrt.shell.text.math.IndexPairFormatV1: Parses the provided string and returns its corresponding index pair. **parse(String)** - Method in class sbrt.shell.text.math.IntervalFormatV1: Parses the provided string and returns its corresponding interval. **parse(String)** - Method in class sbrt.shell.text.math.LinCombTermFormatV1: Parses the provided string and returns its corresponding term. **parse(String, String)** - Method in class sbrt.shell.text.math.LinCombTermFormatV1: Parses the provided strings and returns their corresponding term. **parse(String)** - Method in class sbrt.shell.text.math.LinCombTermFormatV2: Parses the provided string and returns its corresponding term. **parse(String, String)** - Method in class sbrt.shell.text.math.LinCombTermFormatV2: Parses the provided strings and returns their corresponding term. **parse(String)** - Method in interface sbrt.shell.text.math.LinearCombFormat: Parses the provided string and returns its corresponding linear combination. **parse(String)** - Method in class sbrt.shell.text.math.LinearCombFormatV1: Parses the provided string and returns its corresponding linear combination. **parse(String)** - Method in class sbrt.shell.text.math.LinearCombFormatV2: Parses the provided string and returns its corresponding linear combination. **parse(String)** - Method in interface sbrt.shell.text.math.LinearCombTermFormat: Parses the provided string and returns its corresponding term. **parse(String)** - Method in interface sbrt.shell.text.math.MathExprFormat: Parses the provided string and returns its corresponding linear combination. **parse(String)** - Method in class sbrt.shell.text.math.MatrixElementFormatV1: Parses the provided string and returns its corresponding matrix element. **parse(String)** - Method in class sbrt.shell.text.math.SenseFormat: Parses the provided string and returns its corresponding object. **parse(String)** - Method in class sbrt.shell.text.NonEmptyStringFormat: Returns a string equal to the one provided. **parse(String)** - Method in interface sbrt.shell.text.Parser: Parses the provided string and returns its corresponding object. **parse(String)** - Method in interface sbrt.shell.text.PercentFormat: Parses the provided string and returns its corresponding percentage. **parse(String)** - Method in class sbrt.shell.text.PercentFormatVersion1: Parses the provided string and returns its corresponding percentage. **parse(String)** - Method in class sbrt.shell.text.SimpleListFormat: Parses the provided string and returns its corresponding collection of elements as a list. **parse(String)** - Method in class sbrt.shell.text.SimpleSetFormat: Parses the provided string and returns its corresponding collection of elements as a set. **parse(String)** - Method in class sbrt.shell.text.StringFormat: Returns a string equal to the one provided. **parse(String)** - Method in class sbrt.shell.text.TrueFalseFormat: Parses the provided string and returns its corresponding boolean value. **parse(String)** - Method in class sbrt.shell.text.YesNoFormat: Parses the provided string and returns its corresponding boolean value. **parseAllKeys(String)** - Method in class sbrt.shell.text.AbstractMapFormat: Parses the provided string and returns its corresponding set of keys. **parseAllKeys(String)** - Method in class sbrt.shell.text.fba.FbaOptFileLineFormatV1: Parses the provided string and returns its corresponding set of keys. **parseAllKeys(String)** - Method in interface sbrt.shell.text.MapFormat: Parses the provided string and returns its corresponding set of keys. **parseAllPairs(String, String)** - Method in class sbrt.shell.text.AbstractMapFormat: Parses the provided set of keys and list of values and returns a corresponding map of variable-value pairs. **parseAllPairs(String, String)** - Method in class sbrt.shell.text.fba.FbaOptFileLineFormatV1: Parses the provided set of keys and list of values and returns a corresponding map of variable-value pairs. **parseAllPairs(String, String)** - Method in interface sbrt.shell.text.MapFormat: Parses the provided set of keys and list of values and returns a corresponding map of key-value pairs. **parseAllValues(String)** - Method in class sbrt.shell.text.AbstractMapFormat: Parses the provided string and returns its corresponding list of values. **parseAllValues(LinkedHashSet<? extends K>, String)** - Method in class sbrt.shell.text.AbstractMapFormat: Parses the provided string of values and returns a corresponding map of variable-value pairs using the provided set of keys. **parseAllValues(String)** - Method in class sbrt.shell.text.fba.FbaOptFileLineFormatV1: Parses the provided string and returns its corresponding list of values. **parseAllValues(LinkedHashSet<? extends String>, String)** - Method in class sbrt.shell.text.fba.FbaOptFileLineFormatV1: Parses the provided string of values and returns a corresponding map of variable-value pairs using the provided set of keys. **parseAllValues(String)** - Method in interface sbrt.shell.text.MapFormat: Parses the provided string and returns its corresponding list of values. **parseAllValues(LinkedHashSet<? extends K>, String)** - Method in interface sbrt.shell.text.MapFormat: Parses the provided string of values and returns a corresponding map of key-value pairs using the provided set of keys. **parseCorrelator(String)** - Static method in class sbrt.shell.mng.stat.CorrelationManager: Returns the correlation computer corresponding to the provided string value. **parseCorrelator(String)** - Static method in class sbrt.shell.mng.stat.KendallTauManager: Returns the Kendall's Tau statistic computer corresponding to the provided string value. **parseInt(String)** - Static method in class sbrt.shell.mng.AbstractProcessManager: Parses the provided string as a `int`. **parseKey(String)** - Method in class sbrt.shell.text.AbstractMapFormat: Parses the provided string and returns its corresponding key. **parseKey(String)** - Method in class sbrt.shell.text.ColonMapEntryFormatV1: Parses the provided string representation of a key object. **parseKey(String)** - Method in class sbrt.shell.text.EqualsMapEntryFormatV1: Parses the provided string representation of a key object. **parseKey(String)** - Method in class sbrt.shell.text.fba.FbaOptFileLineFormatV1: Parses the provided string and returns its corresponding key. **parseKey(String)** - Method in interface sbrt.shell.text.MapEntryFormat: Parses the provided string and returns its corresponding key. **parseKey(String)** - Method in interface sbrt.shell.text.MapFormat: Parses the provided string and returns its corresponding key. **parseLong(String)** - Static method in class sbrt.shell.mng.AbstractProcessManager: Parses the provided string as a `long`. **parsePair(String)** - Method in class sbrt.shell.text.AbstractMapEntryFormat: Parses the provided map entry string, and returns its corresponding map entry. **parsePair(String)** - Method in class sbrt.shell.text.AbstractMapFormat: Parses the provided map entry string, and returns its corresponding map entry. **parsePair(String)** - Method in class sbrt.shell.text.fba.FbaOptFileLineFormatV1: Parses the provided map entry string, and returns its corresponding map entry. **parsePair(String)** - Method in interface sbrt.shell.text.MapEntryFormat: Parses the provided string and returns a corresponding map entry. **parsePair(String)** - Method in interface sbrt.shell.text.MapFormat: Parses the provided string and returns a corresponding map entry. **parsePrimitive(String)** - Method in interface sbrt.shell.text.BooleanFormat: Parses the provided string and returns its corresponding boolean value. **parsePrimitive(String)** - Method in interface sbrt.shell.text.DoubleFormat: Parses the provided string and returns its corresponding double precision number. **parsePrimitive(String)** - Method in class sbrt.shell.text.DoubleFormatV1: Parses the provided string and returns its corresponding double precision number. **parsePrimitive(String)** - Method in class sbrt.shell.text.DoubleFormatV2: Parses the provided string and returns its corresponding double precision number. **parsePrimitive(String)** - Method in interface sbrt.shell.text.IntegerFormat: Parses the provided string and returns its corresponding integer. **parsePrimitive(String)** - Method in class sbrt.shell.text.IntegerFormatV1: Parses the provided string and returns its corresponding integer. **parsePrimitive(String)** - Method in interface sbrt.shell.text.LongFormat: Parses the provided string and returns its corresponding long. **parsePrimitive(String)** - Method in class sbrt.shell.text.LongFormatV1: Parses the provided string and returns its corresponding long. **parsePrimitive(String)** - Method in interface sbrt.shell.text.math.CoeffFormat: Parses the provided string and returns its corresponding coefficient. **parsePrimitive(String)** - Method in class sbrt.shell.text.math.CoeffFormatV1: Parses the provided string and returns its corresponding coefficient. **parsePrimitive(String)** - Method in class sbrt.shell.text.math.CoeffFormatV2: Parses the provided string and returns its corresponding coefficient. **parsePrimitive(String)** - Method in interface sbrt.shell.text.PercentFormat: Parses the provided string and returns its corresponding percentage. **parsePrimitive(String)** - Method in class sbrt.shell.text.PercentFormatVersion1: Parses the provided string and returns its corresponding percentage. **parsePrimitive(String)** - Method in class sbrt.shell.text.TrueFalseFormat: Parses the provided string and returns its corresponding boolean value. **parsePrimitive(String)** - Method in class sbrt.shell.text.YesNoFormat: Parses the provided string and returns its corresponding boolean value. **Parser**<T> - Interface in sbrt.shell.text: This interface is used to represent *parsers*. **parseValue(String)** - Method in class sbrt.shell.text.AbstractMapFormat: Parses the provided string and returns its corresponding value. **parseValue(String)** - Method in class sbrt.shell.text.ColonMapEntryFormatV1: Parses the provided string representation of a value object. **parseValue(String)** - Method in class sbrt.shell.text.EqualsMapEntryFormatV1: Parses the provided string representation of a value object. **parseValue(String)** - Method in class sbrt.shell.text.fba.FbaOptFileLineFormatV1: Parses the provided string and returns its corresponding value. **parseValue(String)** - Method in interface sbrt.shell.text.MapEntryFormat: Parses the provided string and returns its corresponding value. **parseValue(String)** - Method in interface sbrt.shell.text.MapFormat: Parses the provided string and returns its corresponding value. **Path**<N> - Class in sbrt.kernel.math.graph\_theory: This class can be used for representing paths in a graph or network. **Path(T[])** - Constructor for class sbrt.kernel.math.graph\_theory.Path: Constructs a path containing the nodes from the provided array, in the order they occur starting from index 0. **Path(Collection<? extends N>)** - Constructor for class sbrt.kernel.math.graph\_theory.Path: Constructs a path containing the nodes from the provided collection, in the order they are returned from the collection's iterator. **PathFinder**<N> - Class in sbrt.kernel.math.graph\_theory.comp: This class is an implemenation of an algorithm for finding all pathways in a directed graph. **PathFinder(DirectedGraph<N>)** - Constructor for class sbrt.kernel.math.graph\_theory.comp.PathFinder: Constructs a new path finder for the provided graph. **PathIdManager** - Class in sbrt.shell.mng.graph\_theory: This class is used to manage simple path identification experiments. **PathIdManager()** - Constructor for class sbrt.shell.mng.graph\_theory.PathIdManager: **PathIdProcess**<N> - Class in sbrt.kernel.math.graph\_theory.proc: This class is used to identify all the simple paths in a directed graph. **PathIdProcess()** - Constructor for class sbrt.kernel.math.graph\_theory.proc.PathIdProcess: Constructs a new path identification experiment. **paths(T, S)** - Method in class sbrt.kernel.math.graph\_theory.comp.PathFinder: Returns the number of paths between the provided source and sink nodes. **paths()** - Method in class sbrt.kernel.math.graph\_theory.comp.PathFinder: Returns the total number of paths. **paths(Collection<? extends N>, Collection<? extends N>)** - Method in class sbrt.kernel.math.graph\_theory.comp.PathFinder: Returns the number of paths between the provided source and sink nodes. **PEARSONS\_R** - Static variable in class sbrt.shell.mng.stat.CorrelationManager: The value used to denote a Pearson's r correlation coefficient. **PERCENT** - Static variable in class sbrt.shell.mng.AbstractProcessManager: The default format used for percentages. **PERCENT** - Static variable in class sbrt.shell.text.SpecialChars: % **PERCENT\_COMPLETED** - Static variable in class sbrt.shell.mng.AbstractProcessManager: The prefix used to denote the completed percentage of an operation. **PercentFormat** - Interface in sbrt.shell.text: This interface is used to represent a format for percentages. **PercentFormatVersion1** - Class in sbrt.shell.text: This class is a concrete implementation of `PercentFormat`. **PercentFormatVersion1()** - Constructor for class sbrt.shell.text.PercentFormatVersion1: Constructs a new percent format. **PercentProgressMonitor** - Class in sbrt.kernel.util: This class is used to monitor a process whose progress can be measured as a percentage. **PercentProgressMonitor()** - Constructor for class sbrt.kernel.util.PercentProgressMonitor: Constructs a new percentage-based progress monitor. **PercentProgressMonitor(int)** - Constructor for class sbrt.kernel.util.PercentProgressMonitor: Constructs a new percentage-based progress monitor. **PIPE** - Static variable in class sbrt.shell.text.SpecialChars: | **PipeListFormat**<E> - Class in sbrt.shell.text: This class is used to define a format for pipe-delimited lists. **PipeListFormat(SimpleFormat<E>)** - Constructor for class sbrt.shell.text.PipeListFormat: Constructs a new pipe-delimited list format with the provided element format. **PipeListFormat(Formatter<E>, Parser<E>)** - Constructor for class sbrt.shell.text.PipeListFormat: Constructs a new pipe-delimited list format with the provided element format. **PipeSetFormat**<E> - Class in sbrt.shell.text: This class is used to define a format for pipe-delimited sets. **PipeSetFormat(SimpleFormat<E>)** - Constructor for class sbrt.shell.text.PipeSetFormat: Constructs a new pipe-delimited set format with the provided element format. **PipeSetFormat(Formatter<E>, Parser<E>)** - Constructor for class sbrt.shell.text.PipeSetFormat: Constructs a new pipe-delimited set format with the provided element format. **PLANES\_FILE** - Static variable in class sbrt.shell.mng.geom.CdHarManager: The keyword used to denote the name of the file containing the collection of sets. **plastic()** - Method in class sbrt.kernel.fba.proc.FluxPlasticityProcess.Results: Returns the number of plastic fluxes. **plugIn(Map<?, ? extends LinearComb<? extends V>>)** - Method in class sbrt.kernel.math.HashLinearComb: Plugs in the provided linear combinations to produce a new linear combination. **plugIn(Map<?, Double>, Map<?, ? extends LinearComb<? extends V>>)** - Method in class sbrt.kernel.math.HashLinearComb: Plugs in the provided numerical values and linear combinations to produce a new linear combination. **plugIn(Object, double)** - Method in class sbrt.kernel.math.HashLinearComb: Plugs the provided numerical value into the provided variable to produce a new linear combination. **plugIn(Object, LinearComb<? extends V>)** - Method in class sbrt.kernel.math.HashLinearComb: Plugs the provided linear combination into the provided variable to produce a new linear combination. **plugIn(Object, LinearComb<? extends V>)** - Method in interface sbrt.kernel.math.LinearComb: Assigns the value of the provided variable to the provided linear combination. **plugIn(Object, double)** - Method in interface sbrt.kernel.math.LinearComb: Assigns the value of the provided variable to the provided constant. **plugIn(Map<?, ? extends LinearComb<? extends V>>)** - Method in interface sbrt.kernel.math.LinearComb: Plugs in the provided linear combinations to produce a new linear combination. **plugIn(Map<?, Double>, Map<?, ? extends LinearComb<? extends V>>)** - Method in interface sbrt.kernel.math.LinearComb: Plugs in the provided numerical values and linear combinations to produce a new linear combination. **plugInNumbers(Map<?, Double>)** - Method in class sbrt.kernel.math.HashLinearComb: Plugs in the provided numerical values to produce a new linear combination. **plugInNumbers(Map<?, Double>)** - Method in interface sbrt.kernel.math.LinearComb: Plugs in the provided numerical values to produce a new linear combination. **plugInNumbers(Map<?, Double>)** - Method in interface sbrt.kernel.math.MathExpr: Plugs in the provided numerical values to produce a new mathematical expression. **plugInNumbers(Map<?, Double>)** - Method in interface sbrt.kernel.math.Polynomial: Plugs in the provided numerical values to produce a new polynomial. **PLUS** - Static variable in class sbrt.shell.text.SpecialChars: + **POINTS\_PER\_POINT** - Static variable in class sbrt.shell.mng.geom.CdHarManager: The keyword used to denote the number of points generated for each point returned. **Polynomial**<V> - Interface in sbrt.kernel.math: This interface is used to represent polynomials. **Polynomial.Term**<V> - Interface in sbrt.kernel.math: This interface is used to represent the terms of a polynomial. **POT\_ACTIVE\_FILE\_NAME** - Static variable in class sbrt.shell.mng.fba.FluxPlasticityManager: The keyword used to denote the name of the output file for the names of potentially-active reactions. **potentiallyActive()** - Method in class sbrt.kernel.fba.proc.FluxPlasticityProcess.Results: Returns the number of fluxes that are potentially active. **power(Object)** - Method in class sbrt.kernel.math.AbstractLinearComb.Term: Returns the power of the provided variable in this term. **power(Object)** - Method in interface sbrt.kernel.math.Polynomial.Term: Returns the power of the provided variable in this term. **predecessors(Object)** - Method in class sbrt.kernel.math.graph\_theory.DirectedGraph: Returns the number of predecessors of the provided node. **print(Collection<?>)** - Static method in class sbrt.kernel.util.Util: Prints the elements of the provided collection to `System.out`, one per line. **printBasicInfo(Fluxome, PrintWriter)** - Static method in class sbrt.shell.mng.fba.NetworkInfoManager: Prints basic information about the provided fluxome. **printFluxomeInfo(Fluxome, PrintWriter)** - Static method in class sbrt.shell.mng.fba.FbaManager: Prints basic information about the provided fluxome. **printInput(ManagerInput<String, String>, PrintWriter)** - Static method in class sbrt.shell.ProcessManagerFactory: Creates a formatted string representation of the provided input and prints it to the provided print writer. **printProgress(Number, PrintWriter)** - Static method in class sbrt.shell.mng.AbstractProcessManager: Prints the provided progress to the provided print writer. **printProgress(Integer, PrintWriter)** - Static method in class sbrt.shell.mng.AbstractProcessManager: Prints the provided progress to the provided print writer. **printProgress(Double, PrintWriter)** - Static method in class sbrt.shell.mng.AbstractProcessManager: Prints the provided progress to the provided print writer. **PROCESS** - Static variable in class sbrt.shell.ProcessManagerFactory: The keyword used to denote a procedure. **PROCESS\_NAME\_FILE** - Static variable in class sbrt.shell.ProcessManagerFactory: The keyword used to denote a procedure name file. **ProcessInputFile** - Class in sbrt.shell.io: This class is used to represent process input files. **ProcessInputFile(String, AppInputFileLineFormat)** - Constructor for class sbrt.shell.io.ProcessInputFile: Constructs a new process input file from the provided arguments. **ProcessInputFile(String)** - Constructor for class sbrt.shell.io.ProcessInputFile: Constructs a new process input file from the provided argument. **ProcessManager**<K,V> - Interface in sbrt.shell.mng: This interface is used to represent process managers. **ProcessManagerFactory** - Class in sbrt.shell: This class is used create `ProcessManager` objects. **ProcessRunner** - Class in sbrt: A class to encapsulate the execution of processes. **ProcessRunner()** - Constructor for class sbrt.ProcessRunner: **ProgramSolver**<R,C> - Interface in sbrt.kernel.math.optimization: This interface is used to represent program solvers, as in linear or quadratic program solvers. **ProgramSolverException** - Exception in sbrt.kernel.math.optimization: This class is used to wrap exceptions thrown by program solver packages. **ProgramSolverException(Throwable)** - Constructor for exception sbrt.kernel.math.optimization.ProgramSolverException: Constructs a wrapped program solver exception. **ProgramSolverException(String, Throwable)** - Constructor for exception sbrt.kernel.math.optimization.ProgramSolverException: Constructs a wrapped program solver exception. **ProgramSolverFactory** - Class in sbrt.shell: This class is used to create `ProgramSolver` objects. **ProgramSolverStatus** - Enum in sbrt.kernel.math.optimization: This enum type is used to indicate the status of a program solver. **ProgressMonitor** - Interface in sbrt.kernel.util: This interface is used to represent progress monitors. **PROTEIN\_FILE\_NAME** - Static variable in class sbrt.shell.mng.fba.PalssonSbmlReaderManager: Used to denote the name of the file to which protein-reaction relationships should be written. **PROTEIN\_FILE\_NAME** - Static variable in class sbrt.shell.mng.fba.PalssonSbmlTranslaterManager: Used to denote the name of the file to which protein-reaction relationships should be written. **put(K, V)** - Method in class sbrt.kernel.util.UnmodifiableMap: See Map.put(Object, Object). **putAll(Map<? extends K, ? extends V>)** - Method in class sbrt.kernel.util.UnmodifiableMap: See Map.putAll(Map).

---


|  |  |  |
| --- | --- | --- |
| **Overview** \| Package \| Class \| Use \| **Tree** \| **Deprecated** \| **Index** \| **Help** | |  |
| **PREV LETTER**   **NEXT LETTER** | **FRAMES**    **NO FRAMES**     **All Classes** |  |


A B C D E F G H I J K L M N O P Q R S T U V W X Y Z 

---
